# Two-dimensional transition metal carbides as supports for tuning the chemistry of catalytic nanoparticles

**DOI:** 10.1038/s41467-018-07502-5

**Published:** 2018-12-10

**Authors:** Zhe Li, Liang Yu, Cory Milligan, Tao Ma, Lin Zhou, Yanran Cui, Zhiyuan Qi, Nicole Libretto, Biao Xu, Junwei Luo, Enzheng Shi, Zhenwei Wu, Hongliang Xin, W. Nicholas Delgass, Jeffrey T. Miller, Yue Wu

**Affiliations:** 1 Department of Chemical and Biological Engineering, Iowa State University, Ames, IA 50011 USA; 2 Department of Chemical Engineering, Virginia Polytechnic Institute and State University, Blacksburg, VA 24061 USA; 3 Davison School of Chemical Engineering, Purdue University, West Lafayette, IN 47907 USA; 4 Department of Energy, Ames Laboratory, Ames, IA 50011 USA; 5 Department of Chemistry, Iowa State University, Ames, IA 50011 USA; 6 Present Address: Department of Chemical and Engineering, Stanford University, Stanford, California, 94305 USA

## Abstract

Supported nanoparticles are broadly employed in industrial catalytic processes, where the active sites can be tuned by metal-support interactions (MSIs). Although it is well accepted that supports can modify the chemistry of metal nanoparticles, systematic utilization of MSIs for achieving desired catalytic performance is still challenging. The developments of supports with appropriate chemical properties and identification of the resulting active sites are the main barriers. Here, we develop two-dimensional transition metal carbides (MXenes) supported platinum as efficient catalysts for light alkane dehydrogenations. Ordered Pt_3_Ti and surface Pt_3_Nb intermetallic compound nanoparticles are formed via reactive metal-support interactions on Pt/Ti_3_C_2_T_*x*_ and Pt/Nb_2_CT_*x*_ catalysts, respectively. MXene supports modulate the nature of the active sites, making them highly selective toward C–H activation. Such exploitation of the MSIs makes MXenes promising platforms with versatile chemical reactivity and tunability for facile design of supported intermetallic nanoparticles over a wide range of compositions and structures.

## Abstract (web summary)

The performance of supported metal nanoparticle catalysts can be tailored by metal-support interactions, but their use in catalyst design is still challenging. Here, the authors develop two-dimensional transition metal carbides as platforms for designing intermetallic compound catalysts that are efficient for light alkane dehydrogenations.

## Introduction

Metal nanoparticles (NPs) are widely used as heterogeneous catalysts and in electrochemical applications^1–3^. The performance of the supported metal NPs, including the rate, selectivity and stability, can be tailored by controlling their interactions with the supports^4–7^. These metal-support interactions (MSIs) have been found to modify the geometric and electronic structures of active sites^8,9^, and, not surprisingly, the chemical properties of the supports are crucial to the modifications^10^. Nonetheless, rational design of supported catalysts through MSIs is often arduous, especially when the supports undergo structural changes under reaction conditions^11^. A classic example is the encapsulation of metal active sites by reducible oxide-support overlayers, which was designated by Tauster et al.^12^ as the strong-metal support interaction (SMSI) in 1978. In the SMSI state the active metal sites on the NPs are covered due to the migration of suboxide species, which renders the loss of adsorption capability of the NPs^12,13^. Supports with ideal chemical tunability and reactivity are clearly a key to harnessing the potential promotional effects of MSIs on supported metal NP catalysts, but their development remains a grand challenge.

Two-dimensional (2D) early transition metal carbides, i.e., MXenes, is a burgeoning class of materials with well-defined structures and widely tunable compositions^14^. They have a formula of M_*n*+1_X_*n*_T_*x*_, where M is an early transition metal, X refers to carbon and/or nitrogen, and T stands for surface functional groups^15^. We have recently shown that MXenes are promising supports for nanoparticle (NPs) catalysts and that the presence of noble metal NPs promotes both the removal of surface functional groups and reduction of the M component of the MXene^16^. Reduction of these catalyst supports can lead to targeted delivery of the metal components in the supports to the NPs that contact the support surface. As a result, formation of ordered intermetallic compounds (IMCs) through reactive metal-support interactions (RMSIs) is possible^17^. RMSI refers to a chemical reaction between a metal and the support that induces the formation of bimetallic structures, which is differentiated from the more general SMSI because it is driven by the high thermodynamic stability of the resulting IMCs. MXenes can facilitate this process by having 2D structures with metal carbon bonds (M–C) that are weaker compared to the metal oxygen bonds (M–O) in typical oxide supports. This enhanced chemical reactivity can allow RMSIs to occur at lower temperature and, thus, favor the control of particle size, in contrast to the high temperature reduction required for early transition metal oxides or bulk carbides^18–20^. On MXenes IMCs may be formed that are not possible on traditional oxide and carbide supports and their properties controlled through in situ reduction at moderate temperature.

Here, we report two examples of thermally stable intermetallic NP catalysts prepared via RMSI between platinum and MXenes. A complete, full Pt_3_Ti IMC with Cu_3_Au type structure is formed in Pt/Ti_3_C_2_T_*x*_ catalysts. For Pt/Nb_2_CT_*x*_ catalysts, NPs with a surface Pt_3_Nb IMC in the same structure are formed, presumably via a process kinetically controlled by the diffusion of Nb species. These intermetallic structures have not been previously reported for Pt NPs catalysts supported by oxides and bulk carbides of Ti and Nb. The strong intermetallic bonds in these structures offer compositional and electronic modification of the actives sites. The result, in this case is that the catalysts become highly selective for light alkane dehydrogenation (LADH), a reaction in renewed interest due to shale gas boom^21^. Such reactive interaction is generally applicable between platinum NPs and MXene families. The Pt-M (M refers to early transition metals) IMCs are famous for their thermal stability with high enthalpy of formation, but their preparation through co-reduction is challenging as early transition metals are oxyphilic^22,23^. Thus, MXenes pave an avenue for facile design of Pt-M NPs with a broad range of compositions and structures that are intractable to synthesize by traditional methods.

## Results

### Two-dimensionality of the MXene supports

**Fig. 1:**
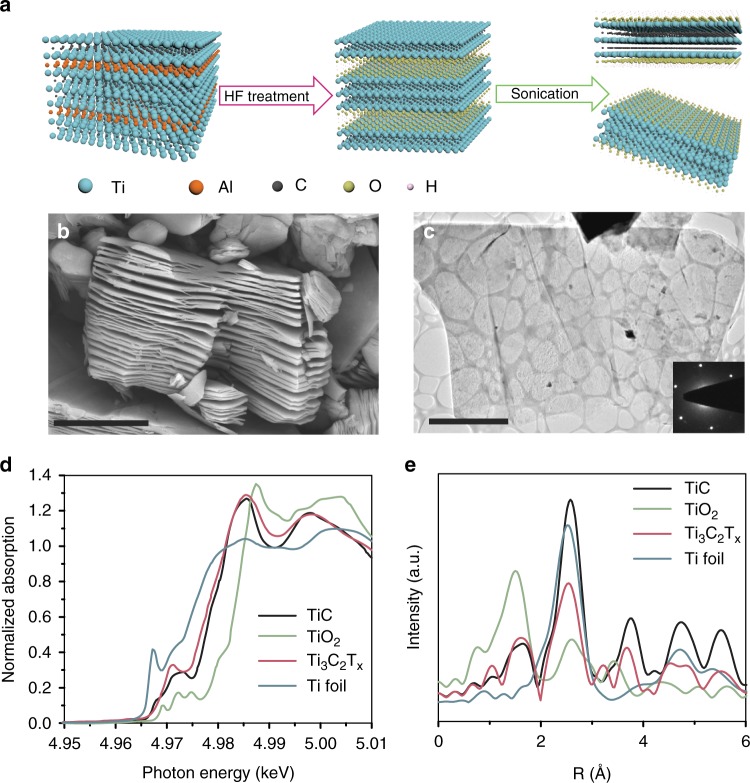
Characterization of Ti_3_C_2_T_*x*_ MXene support. **a** Schematic of Ti_3_C_2_T_*x*_ MXene preparation. **b** SEM image of Ti_3_C_2_T_*x*_ MXene, the scale bar corresponds to 3 µm. **c** TEM image of Ti_3_C_2_T_*x*_ MXene nanosheets. Inset represents the selected area electron diffraction (SAED) pattern showing hexagonal basal plane symmetry of Ti_3_C_2_T_*x*_ MXene. The scale bar corresponds to 2 µm. **d** XANES spectra of the Ti_3_C_2_T_*x*_ compared to references including Ti metal foil, TiO_2_ and TiC. **e** Magnitude of the Fourier transform of the k^2^ weighted EXAFS of the Ti_3_C_2_T_*x*_ compared to bulk TiC

The two MXene supports, Ti_3_C_2_T_*x*_ and Nb_2_CT_*x*_, were prepared by a chemical exfoliation process reported by the literature^14,24^. Briefly, Ti_3_AlC_2_ and Nb_2_AlC compounds (MAX) were treated with HF to extract the aluminum layers and exfoliate the 2D early transition metal carbides (Fig. 1a). In the X-ray diffraction (XRD) patterns (Supplementary Figs. 1a, b), the shift of (002) peaks and the disappearance of the most intense nonbasal plane diffraction peaks of the MAX phases at 2*θ* ≈  39° indicate that the MAX phases are converted to MXenes with increased c lattice parameters after the HF exfoliation. The scanning electron microscopy (SEM) images (Fig. 1b, Supplementary Fig. 1c) display the typical accordion-like morphology of MXenes that suggests the exfoliation of individual grains along the basal planes. Dimethyl sulfoxide (DMSO) and tetrapropylammonium hydroxide (TPAOH) were employed as intercalants to delaminate Ti_3_AlC_2_ and Nb_2_CT_*x*_ MXenes, respectively^25,26^. With the help of sonication, thin layers of MXenes nanosheets that are electron transparent can be obtained (Fig. 1c, Supplementary Fig. 1d).

The chemical nature of the MXenes was investigated by X-ray absorption spectroscopy (XAS). Figure 1d shows that the Ti K edge X-ray absorption near edge spectroscopy (XANES) spectrum of Ti_3_C_2_T_*x*_ has similar shape compared to that of bulk TiC rather than that of Ti foil or TiO_2_. The edge energy of Ti_3_C_2_T_*x*_ (4967.2 eV) is close to that of TiC (4967.1 eV), which is between the energies of Ti foil (4966.4 eV) and TiO_2_ (4968.6 eV), indicating its carbide nature. The extended X-ray absorption fine structure (EXAFS) spectra (Fig. 1e) compare the local coordination of the Ti atoms in the Ti_3_C_2_T_*x*_ MXene to its bulk counterpart (TiC). Ti_3_C_2_T_*x*_ shows first-shell scattering (Ti-C/O/F) similar to that of TiC but with second-shell scattering (Ti-C-Ti) lower than that of TiC, consistent with the reduced dimensionality of the Ti_3_C_2_T_*x*_ MXene and corresponding high portion of surface Ti atoms. Similar results were obtained for Nb_2_CT_*x*_ by Nb K edge XAS in our previous work^16^.

### Characterizations of MXenes supported nanoparticles

Pt was loaded on the two MXene supports via incipient-wetness impregnation (IWI) as reported previously^16^. The Pt/MXene catalysts were further reduced with 5% H_2_/N_2_ at 550 °C, which is within the typical temperature range of LADH reactions^27^. High angle annular dark field scanning transmission electron microscopy (HAADF-STEM) shows that small NPs form on both catalysts. The average particle diameters are about 6 ± 3.2 nm and 2.6 ± 0.7 nm for Pt/Ti_3_C_2_T_*x*_ and Pt/Nb_2_CT_*x*_ catalysts, respectively. The MXene supports enable dispersion of NPs without apparent agglomeration (Supplementary Figs. 2, 3).

**Fig. 2:**
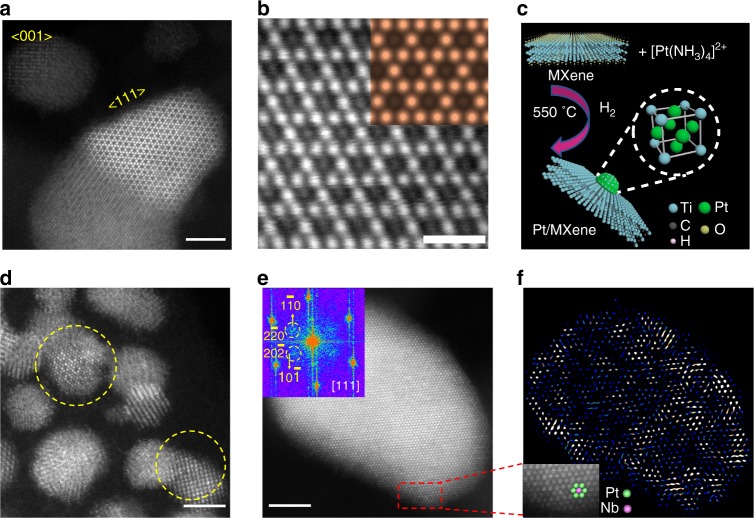
Microscopy characterizations of 1% Pt/MXene catalysts. **a** Representative HAADF-STEM image of 1% Pt/Ti_3_C_2_T_*x*_ catalyst. **b** (111) surface of Pt_3_Ti NP. Inset is a simulated STEM image of Pt_3_Ti (111) surface. The simulated image is in good agreement with the experimental result. **c** Schematic illustration of RMSI in Pt/MXene catalysts and the structure of L1_2_-ordered intermetallic Pt_3_Ti. **d** Representative HAADF-STEM image of 1% Pt/Nb_2_CT_*x*_ catalyst. **e** A Pt-Nb NP viewed along [111], inset is the FFT pattern of the NP. **f** IFFT pattern of the NP in Fig. 2e, inset is an enlarged image showing the super lattice of the NP. Scale bars: **a**, **d**, **e** 2 nm, and **b** 500 pm

The compositions of the NPs were first investigated by energy dispersive spectroscopy (EDS) elemental mapping. The signals of Pt and Ti or Nb overlap over all the NPs (Supplementary Figs. 4, 5), suggesting migrations of M to the Pt NPs. Aberration-corrected HAADF-STEM was utilized to further characterize the structures of Pt-Ti and Pt-Nb NPs. Figure 2a shows an image representative of the NPs supported by Ti_3_C_2_T_*x*_ MXene. The NP in the center of the figure is viewed along the [111] zone axis, whereas another NP in the upper left corner is viewed along the [001] zone axis. Two different types of atoms can be clearly differentiated due to the Z-contrast in high angle annular dark field (HAADF) imaging. The bright dots are characteristic of heavier Pt atoms, while the dimmer ones correspond to Ti. Along the [111] axis of a NP, the projected unit cells are composed of periodic hexagonal arrays of Pt atoms that surround Ti atoms at the center of the hexagons (Fig. 2b), which indicates a specific L1_2_ type symmetry and is consistent with formation of the Cu_3_Au type Pt_3_Ti IMC (Fig. 2c)^22^. The experimental HAADF-STEM image shows good agreement with the simulated (111) surface of L1_2_ ordered Pt_3_Ti nanostructures (inset of Fig. 2b). The Pt/Ti ratio is estimated by EDS elemental mapping on a NP hanging over the vacuum to avoid the Ti signals from the Ti_3_C_2_T_*x*_ MXene support and give a value of the molar ratio of Pt to Ti equal to 3.55 (Supplementary Fig. 6), which is close to the theoretical ratio of Pt_3_Ti alloy within error.

The ordered intermetallic structure of Pt-Nb NPs can also be directly observed (circled with dash lines) despite the small particle sizes (2.6 ± 0.7 nm) (Fig. 2d). The fcc structure is identified by two NPs viewed along [111] and [001] axis, respectively (Fig. 2e, Supplementary Fig. 7). The inset of Fig. 2e shows the fast Fourier transform (FFT) pattern of the NP viewed along [111] zone, where the signals of ($1\overline 1 0$) and ($10\overline 1$) supper-lattice are present. Since the unique super periods are present only in the structurally ordered intermetallic phase and are absent in the disordered alloy phase, ordering in the NP is confirmed. Moreover, the enlarged image of the NP (inset of Fig. 2f) shows a dimmer Nb atom surrounded by six Pt atoms in a hexagonal pattern, again implying a local L1_2_ ordering. The intermetallic phase, however, does not form over the whole NP. The distribution of the Pt-Nb intermetallic order in the NP is demonstrated by the contrast variation of the inverse fast Fourier transform (IFFT) image in Fig. 2f. The IFFT pattern shows that the ordered Pt-Nb phase preferentially populates the edge of the NP versus the inner core. This is consistent with previous studies reporting that the diffusion of the second metallic species into the noble metal lattice (from the NP surface) played a pivotal role in the formations of intermetallic NPs^28–30^.

**Fig. 3:**
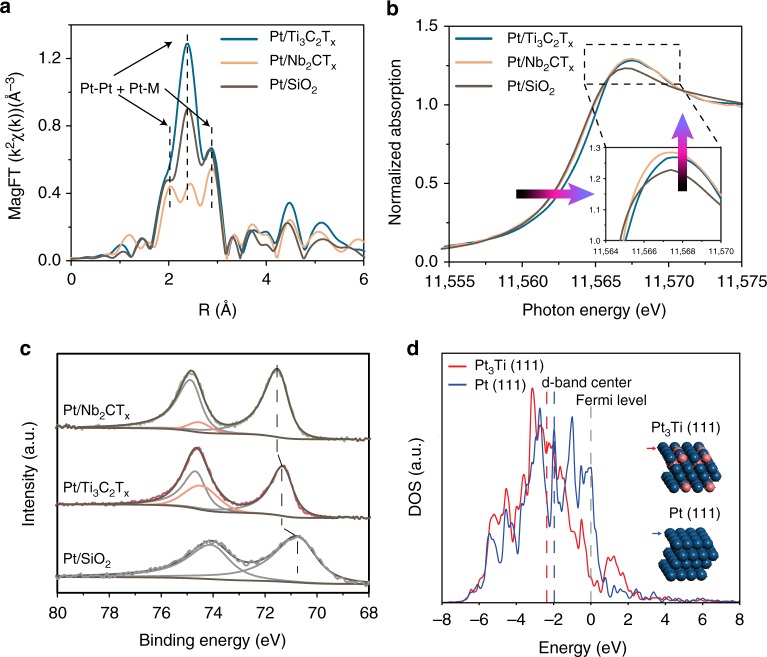
In situ Spectroscopy characterization of Pt/MXenes catalysts. **a** Magnitude of the Fourier Transform of the k^2^ weighted Pt L_III_ edge in situ EXAFS of the Pt/Ti_3_C_2_T_*x*_ and Pt/Nb_2_CT_*x*_ catalyst after reduction at 550 °C in H_2_ compared to Pt/SiO_2._ **b** The Pt L_III_ edge in situ XANES spectra of the Pt/Ti_3_C_2_T_*x*_ and Pt/Nb_2_CT_*x*_ catalyst after reduction at 550 °C in H_2_ compared to Pt/SiO_2_. **c** XPS spectra of Pt/Ti_3_C_2_T_*x*_, Pt/Nb_2_CT_*x*_ and Pt/SiO_2_ reduced at 550 °C by H_2_ in a spectrometer side chamber and not exposed to air. **d** DFT calculated projected density of states (DOS) for the 5*d* orbitals of Pt in the top-layer Pt_3_Ti (111) and Pt (111)

To investigate the altered Pt chemical environment resulting from the RMSI between Pt and MXenes, the Pt/MXene catalysts were studied by in situ XAS. A Pt/SiO_2_ catalyst with an average particle size of 1.8 ± 0.6 nm was also prepared as a reference (Supplementary Fig. 8). The EXAFS spectra of both in situ reduced catalysts (Fig. 3a) show metal-metal scattering significantly different from the typical three-peak pattern characteristic of monometallic Pt NPs on SiO_2_. For Pt/Ti_3_C_2_T_*x*_, the central peak of the magnitude of Fourier transform EXAFS has a higher intensity due to the in-phase constructive interference between Pt-Ti and Pt-Pt scattering, while the opposite is observed on Pt/Nb_2_CT_*x*_ because Pt-Nb and Pt-Pt are out-of-phase. Quantitative fitting (Supplementary Fig. 9, Supplementary Table 1) of the EXAFS gives the following average coordination numbers (CNs) and bond distances: 6.6 Pt-Pt bond and 3.4 Pt-Ti bond both at 2.75 Å for the Pt/Ti_3_C_2_T_*x*_ catalyst and 6.7 Pt-Pt bond at 2.77 Å and 1.8 Pt-Nb bond at 2.75 Å for the Pt/Nb_2_CT_*x*_ catalyst. The EXAFS of both catalysts confirms the presence of bimetallic NPs. For an ideal bulk L1_2_ type Pt_3_M intermetallic structure, the bond distances are the same for the Pt-Pt and Pt-M paths and the ratio of Pt-Pt CN over Pt-M CN is 2. Thus, the XAS results for Pt/Ti_3_C_2_T_*x*_ indicate the formation of L1_2_ type Pt_3_M structures, which is concordant with those of STEM. For the Pt/Nb_2_CT_*x*_, the Pt-Pt/Pt-Nb CN ratio is much greater than 2 and their bond distances are slightly different, consistent with formation of partial/surface L1_2_ ordering in the Pt-Nb NPs as observed by STEM. Together the HAADF-STEM and EXAFS indicate that RMSI occurs in these two MXene supported catalysts, leading to formations of IMCs with tunable compositions and structures. We note for comparison that Pt added similarly to bulk titanium carbide or niobium carbide surfaces did not produce IMCs (Supplementary Figs. 10, 11).

### Electronic structures of Pt/MXene catalysts

In situ XANES at Pt L_III_ edge was conducted on the reduced catalysts to examine the energy of the unoccupied *d* states (Fig. 3b). The XANES energies of the Pt/Ti_3_C_2_T_*x*_ and Pt/Nb_2_CT_*x*_ catalysts are 11564.6 eV and 11564.3 eV, respectively, both higher compared to that of monometallic Pt (11564.0 eV). The whitelines are slightly taller and narrower compared to that of Pt/SiO_2_, corresponding to a change in the energy distribution of the 5*d* unoccupied states. Shifts in electronic band energy level were also detected by XPS (in situ reduced samples). In Fig. 3c, the Pt 4*f*_7/2_ binding energies of Pt/Ti_3_C_2_T_*x*_ and Pt/Nb_2_CT_*x*_ are 71.3 eV and 71.6 eV respectively, which shift to higher values with respect to that of monometallic Pt (70.8 eV). These spectroscopic studies of fully reduced samples indicate that the formation of Pt_3_M intermetallic NPs leads to altered electronic structure of the Pt atoms and that the 5*d* states are shifted to higher energy in Pt/MXenes compared to Pt/SiO_2_.

To better understand the modulated electronic structures of the Pt/MXene catalysts owing to the formations of IMCs, we carried out density functional theory (DFT) calculations of the projected density of states (DOS). Out of the two Pt/MXene catalysts, the Pt/Ti_3_C_2_T_*x*_ catalyst contains full (instead of partial) IMC NPs with the exposure of representative (111) surface planes resolved by aberration-corrected STEM. Therefore, we employed slab models of Pt_3_Ti(111) and Pt(111) surfaces for comparison. As shown in Fig. 3d, the *d* band center of Pt(111) is located at −1.97 eV relative to the Fermi level, whereas that of Pt_3_Ti(111) downshifts to −2.37 eV due to a strong Pt-Ti *d*-*d* orbital coupling. Similar shifts of *d* band center to a lower level were also reported in other Pt-M systems, where M is Co, Fe, Ni, Ce^31^. Additionally, ~1 eV above the Fermi level, the DOS calculation also shows an increased intensity for Pt_3_Ti(111) (Fig. 3d) compared to Pt(111), which is consistent with the in situ XANES results showing higher energy and intensity of the whiteline of the Pt/Ti_3_C_2_T_*x*_ catalyst compared to Pt/SiO_2_. A recent study combining DFT calculations and an emerging technique, in situ resonant inelastic X-ray scattering (RIXS), reported that formation of IMCs leads to a downward shift of the *d* band center as well as an upward shift of the energy of the unoccupied 5*d* states^32^, which agrees with our results.

### Catalytic performance and free energy calculations

**Fig. 4:**
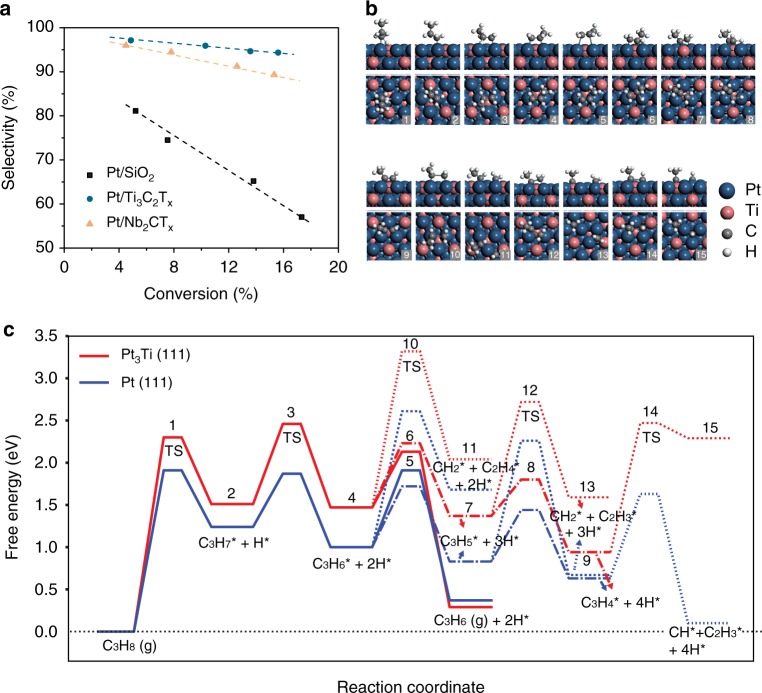
Catalytic performance and DFT calculation of Pt/MXene catalysts. **a** Plots of conversion vs. selectivity for propane dehydrogenation measured in 200 cm^3^ min^−1^ of 2.5% C_3_H_8_, 2.5% H_2_ balanced in N_2_ at 1.5 atm and 550 °C for Pt/Ti_3_C_2_T_*x*_, Pt/Nb_2_CT_*x*_, and Pt/SiO_2_ catalyst. **b** Snapshots of optimized structures as numbered in **c** from side and top view angles (H* is not shown). **c** DFT-calculated free energy diagram of relevant (side-)reaction steps in propane dehydrogenation on Pt_3_Ti (111) and Pt (111) surfaces. The dotted lines denote the C–C cracking reactions of C_3_H_6_*, C_3_H_5_*, and C_3_H_4_*, generating CH_2_* + C_2_H_4_*, CH_2_* + C_2_H_3_*, and CH* + C_2_H_3_*, respectively. The dash-dot lines denote the further dehydrogenation of C_3_H_6_* to C_3_H_5_*, and C_3_H_5_* to C_3_H_4_*

According to *d* band chemisorption theory, shifts to lower energy of *d* band center will reduce the surface adsorption reactivity^33^ and directly affect the chemistry of catalysts^34^. LADH reactions are sensitive to the energy level of *d* band electrons of the catalysts surface^21^, thus were used as probes to evaluate effects of the in situ formation of Pt_3_M intermetallic NPs on the catalytic performance. All the catalysts were pretreated and tested under the same conditions summarized in Methods. The product selectivity of different catalysts was compared between 0 and 20% light alkane conversions. For both dehydrogenation of propane (Fig. 4a) and isobutane (Supplementary Fig. 12), Pt/MXene catalysts are much more selective than Pt/SiO_2_ at the same conversion. For example, when the conversion of propane is 15%, Pt/SiO_2_ is 60% selective to propylene, while those of Pt/Ti_3_C_2_T_*x*_ and Pt/Nb_2_CT_*x*_ are ~95% and ~90%, respectively (Fig. 4a). Though intermetallic catalysts have larger particle size compared to the reference monometallic Pt catalyst, the improvement in their catalytic performance can be attributed to the formation of intermetallic structure rather than a size effect since larger particles have been reported to give lower selectivity^35,36^. For all the catalysts, the selectivity of dehydrogenation is lower at higher conversion due to the hydrogenolysis side reaction that requires hydrogen. The decrease in selectivity under increasing conversion is reduced on the Pt/Ti_3_C_2_T_*x*_ and Pt/Nb_2_CT_*x*_ catalysts, indicating that the effect of side reactions is less prominent on the intermetallic NPs. The TORs were calculated using the reaction rate per gram of Pt measured under differential conditions and catalyst dispersion estimated from the average particles sizes. For propane dehydrogenation (PDH), the TORs were 0.12 s^−1^, 0.09 s^−1^ and 0.08 s^−1^ for Pt/SiO_2_, Pt/Nb_2_CT_*x*_ and Pt/Ti_3_C_2_T_*x*,_ respectively. The TORs for isobutane dehydrogenation followed a similar trend and were 0.09 s^−1^, 0.06 s^−1^ and 0.06 s^−1^ for Pt/SiO_2_, Pt/Nb_2_CT_*x*_ and Pt/Ti_3_C_2_T_*x*_. These values are similar to the TORs reported for typical LADH catalysts^21^. The evolution of performance with time on-stream for IMC catalysts are also consistent with the monometallic Pt catalyst (Supplementary Fig. 13) as well as previous literature, due to slow deposition of coke^21^. The used Pt/Nb_2_CT_*x*_ and Pt/Ti_3_C_2_T_*x*_ were further characterized by HAADF-STEM to check the stability of the IMCs. The structures of Pt_3_Ti and Pt_3_Nb are preserved in the spent catalysts (Supplementary Figs. 14, 15), indicating that the IMCs NPs were stable under the LADH reaction conditions.

To understand the high olefin selectivity of the intermetallic catalysts for PDH reaction, energy profiles of PDH reaction and possible side reactions were studied by DFT calculations. Snapshots of structures of reaction intermediates and transition states were illustrated in Fig. 4b, with the free energies of the relevant reaction pathways on Pt(111) and Pt_3_Ti(111) surfaces calculated and shown in Fig. 4c. PDH follows a step-wise C–H bond breaking process^37^, which starts with dissociative adsorption of propane forming surface alkyl species (intermediate 2), followed by the scission of a secondary C–H bond generating adsorbed olefins (intermediate 4). Our DFT results show that the free energy changes and barriers of the first two steps on the Pt_3_Ti(111) are higher than those on pure Pt(111), indicating weakened surface adsorption activity of the intermetallic phase consistent with the shifts in the 5*d* DOS indicated by DFT, XANES, and XPS. In the following steps, the adsorbed olefins may undergo desorption, further (deep) dehydrogenation (intermediate 7) or C–C breaking (intermediate 11) processes. The latter two steps are believed to generate the precursors leading to side reactions, i.e., coking and hydrogenolysis^38^.

On Pt(111), the free energy barrier for dehydrogenation of C_3_H_6_* to C_3_H_5_* is 0.19 eV lower than that of propylene desorption, indicating that deep dehydrogenation is more favorable on Pt(111) surface. In contrast, on Pt_3_Ti(111) the C_3_H_6_* desorption is more favored by 0.1 eV in barrier than further dehydrogenation. For direct comparison, the barrier of C_3_H_6_* desorption on Pt_3_Ti(111) (0.66 eV) is 0.25 eV lower than that on Pt(111) (0.91 eV). In addition, the free energy change of C_3_H_6_* desorption on Pt_3_Ti(111) (−1.17 eV) is 0.54 eV more favorable than that on Pt(111) (−0.63 eV). Overall, the introduction of Ti lowers the desorption barrier of C_3_H_6_* to the gas phase (0.66 eV of Pt_3_Ti(111) vs. 0.91 eV of Pt(111)) and increases the energy barrier for deep dehydrogenation. On both surfaces, C–C cracking of dehydrogenated reaction intermediates, e.g., C_3_H_6_*, C_3_H_5_*, and C_3_H_4_*, requires much higher activation energies and thus are less competitive. Nevertheless, on Pt_3_Ti(111) the C–C cracking steps (intermediates 11, 13, and 15) are all endergonic and hence are hindered compared with Pt(111) where C_3_H_5_* and C_3_H_4_* cracking are exergonic and much more favorable. These results rationalize the observed higher selectivity toward propylene for PDH by Pt_3_Ti intermetallic NPs compared to pure Pt.

Our experimental results and DFT calculations show that the Pt_3_Ti intermetallic phase has a lower *d* band center compared with that of monometallic Pt, which results in weaker adsorption of light hydrocarbon species and changes of the relative free energy and barriers of the reaction steps during dehydrogenation and side reactions. Lowering of the olefin desorption barrier to below that of deep dehydrogenation and C–C breaking contributes to the high catalyst selectivity. The same type of calculations were not conducted for the Pt/Nb_2_CT_*x*_ catalyst due to its relatively less well-defined structure. However, similar catalyst electronic structure compared to Pt/Ti_3_C_2_T_*x*_ can be expected according to the in situ X-ray spectroscopy results. The adsorption properties, and reaction energetics are, therefore, also expected to be similar. The fact that the Pt/Nb_2_CT_*x*_ catalyst has a slightly lower selectivity compared to Pt/Ti_3_C_2_T_*x*_ is likely due to the extent of the IMCs formation, i.e., full verses surface IMCs, and differences in electronic effects. These additional subtle catalytic differences demonstrate that the chemical properties of the catalysts are tunable using different MXene materials as the RMSI-active supports.

In summary, this work demonstrates two IMC catalysts selective for LADH achieved by RMSI between Pt NPs and Ti_3_C_2_T_*x*_ and Nb_2_CT_*x*_ MXenes. The intermetallic surface is imaged by atomic resolution HAADF-STEM and its high catalytic selectivity is rationalized by DFT calculations. With MXenes as catalyst supports and through their active interactions with metal NPs, there is an opportunity to explore many new compositions for heterogeneous catalysis in industrial gas-phase reactions as well as electrochemical conversion, with the possibility that the chemical and electronic properties of the resulting catalysts can be tuned over a wider range than what is currently possible using conventional catalyst supports.

## Methods

### Synthesis of Ti_3_AlC_2_ and Nb_2_AlC

The Ti_3_AlC_2_ powder was synthesized by spark plasma sintering (SPS) of TiH_2_/Al/TiC. Commercial powders of titanium (II) hydride, aluminum and titanium (IV) carbide were mixed in a molar ratio of TiH_2_/Al/TiC = 1:1:1.8 in a graphite die coated with boron nitride (BN). Excess Al and less than a full equivalent of TiC were added because a small portion of Al will be lost during high-temperature processing, and carbon deficiencies exist in most Al-containing MAX phases. Then, the material was loaded in a Fuji-211lx SPS system and sintered at 1350 °C under 30 MPa for 1 h. The resulting Ti_3_AlC_2_ was then pulverized and sieved through a 325-mesh screen.

The Nb_2_AlC powder was synthesized by SPS of Nb/Al/graphite mixtures. Commercial powders of niobium, graphite and aluminum were mixed in a molar ratio of Nb:Al:C = 2:1.4:0.9 in a graphite die coated with boron nitride (BN). Then, the material was loaded in a Fuji-211lx SPS system and sintered at 1500 °C under 30 MPa for 1 h. The resulting Nb_2_AlC was then pulverized and sieved through a 400-mesh screen.

### Synthesis of Ti_3_C_2_T_*x*_ and Nb_2_CT

Preparation of Ti_3_C_2_T_*x*_ MXene: Approximately 1 g of the prepared Ti_3_AlC_2_ powder was immersed in 10 mL of 50% aqueous hydrofluoric acid solution stirred with a magnetic bar for approximately 1 days at 35 °C. The resulting MXene suspension was repeatedly washed with deionized water (DI) and centrifuged until the pH reached ~5. The final MXene was dried under vacuum at room temperature and stored in a glove box until usage.

Preparation of Nb_2_CT_*x*_ MXene: Approximately 1 g of the prepared Nb_2_AlC powder was immersed in 10 mL of 50% aqueous hydrofluoric acid (HF) solution stirred with a magnetic bar for approximately 3 days at 55 °C. The resulting MXene suspension was repeatedly washed with deionized water (DI) and centrifuged until the pH reached ~5. The final MXene was dried under vacuum at room temperature and stored in a glove box until usage.

### Catalyst preparation

A monometallic Pt catalyst (2 wt. % Pt supported on Davisil 636 silica gel from Sigma-Aldrich) was synthesized using the incipient wetness impregnation (IWI) method. 0.20 g of tetraammineplatinum nitrate was dissolved in 3 mL of H_2_O.Approximately 30% ammonium hydroxide solution (NH_4_OH, 28% NH_3_ in H_2_O, ≥99.99%, Sigma-Aldrich) was then added to the solution until the pH reached 11. The obtained Pt precursor solution was added dropwise to 5 g of silica and stirred. After drying overnight under vacuum, the sample was calcined at 225 °C for 3 h and reduced at 550 °C in 5% H_2_/N_2_ for 30 min.

Pt supported on Ti_3_C_2_T_*x*_ and on Nb_2_CT_*x*_ were prepared via a similar method. 0.20 g of tetraammineplatinum nitrate Pt(NH_3_)_4_(NO_3_)_2_ were dissolved in 0.5 mL of H_2_O to prepare 1 mol L^−1^ Pt precursor solution. 0.05 mL of such solution was impregnated on fresh Ti_3_C_2_T_*x*_ and Nb_2_CT_*x*_, respectively, prior to dying overnight under vacuum. The obtained catalysts were reduced at 550 °C in 5% H_2_/N_2_ for at least 0.5 h before each catalytic test or characterization.

### LADH kinetics

LADH kinetics measurements were carried out in a quartz fixed-bed reactor with 3/8-inch ID. Catalysts around 0.02–0.15 g were diluted using pure SiO_2_ to achieve a total weight of 1.00 g for testing the performance. Reaction temperature was measured using a thermocouple inserted in a stainless-steel thermocouple well locating at the bottom center of the catalyst bed. Agilent 7890A gas chromatograph system quipped with a flame ionization detector (FID) was employed for analyzing the products. Prior to each measurement, the fresh catalysts were reduced by 5% H_2_/N_2_ (50 cm^3^ min^−1^) for 30 min at 550 °C with the temperature ramping rate of 15 °C min^−1^. Propane dehydrogenation was tested under a reaction atmosphere of 2.5% C_3_H_8_, 2.5% H_2_ balanced with N_2_. The total flow rate of the reactant mixture was 200 cm^3^ min^−1^. After 2 min on-stream, the catalyst selectivity was compared below 20% conversion at 550 °C and turnover rates (TORs, per surface Pt site) were measured under differential condition at conversion below 5%. For iso-butane dehydrogenation, a reaction atmosphere of 2.5% C_3_H_8_, 2.5% H_2_ balanced in N_2_ with a total flow rate of 100 cm^3^ min^−1^ was used. Catalyst performance was measured at 450 °C.

## Electronic supplementary material

Supplementary Information

## Data Availability

The datasets generated during and/or analyzed during the current study are available from the corresponding authors on reasonable request.

## References

[1] Bell AT (2003). The impact of nanoscience on heterogeneous catalysis. Science.

[2] Shekhar M (2012). Size and support effects for the water–gas shift catalysis over gold nanoparticles supported on model Al_2_O_3_ and TiO_2_. J. Am. Chem. Soc..

[3] Huang X (2015). High-performance transition metal-doped Pt_3_Ni octahedra for oxygen reduction reaction. Science.

[4] Matsubu JC (2017). Adsorbate-mediated strong metal-support interactions in oxide-supported Rh catalysts. Nat. Chem..

[5] Kattel S, Liu P, Chen JG (2017). Tuning selectivity of CO_2_ hydrogenation reactions at the metal/oxide interface. J. Am. Chem. Soc..

[6] Zhao ZJ (2017). Importance of metal-oxide interfaces in heterogeneous catalysis: a combined DFT, microkinetic, and experimental study of water-gas shift on Au/MgO. J. Catal..

[7] Mehta P, Greeley J, Delgass WN, Schneider WF (2017). Adsorption energy correlations at the metal-support boundary. ACS Catal..

[8] Li WZ (2013). Stable platinum nanoparticles on specific MgAl_2_O_4_ spinel facets at high temperatures in oxidizing atmospheres. Nat. Commun..

[9] Rao RG (2017). Interfacial charge distributions in carbon-supported palladium catalysts. Nat. Commun..

[10] Cargnello M (2013). Control of metal nanocrystal size reveals metal-support interface role for ceria catalysts. Science.

[11] Willinger MG (2014). A case of strong metal-support interactions: combining advanced microscopy and model systems to elucidate the atomic structure of interfaces. Angew. Chem. Int. Ed..

[12] Tauster S, Fung S, Garten RL (1978). Strong metal-support interactions. Group 8 noble metals supported on titanium dioxide. J. Am. Chem. Soc..

[13] Tauster S, Fung S, Baker R, Horsley J (1981). Strong interactions in supported-metal catalysts. Science.

[14] Naguib M (2011). Two‐dimensional nanocrystals produced by exfoliation of Ti_3_AlC_2_. Adv. Mater..

[15] Naguib M, Mochalin VN, Barsoum MW, Gogotsi Y (2014). 25th anniversary article: MXenes: a new family of two-dimensional materials. Adv. Mater..

[16] Li Z (2018). Reactive metal-support interactions at moderate temperature in two-dimensional niobium-carbide-supported platinum catalysts. Nat. Catal..

[17] Penner S, Armbrüster M (2015). Formation of intermetallic compounds by reactive metal–support interaction: a frequently encountered phenomenon in catalysis. ChemCatChem.

[18] Bernal S (1997). Nanostructural Evolution of a Pt/CeO_2_ catalyst reduced at Increasing temperatures (473–1223 K): a HREM study. J. Catal..

[19] Beard BC, Ross PN (1986). Platinum-titanium alloy formation from high-temperature reduction of a titania-impregnated platinum catalyst: implications for strong metal-support interaction. J. Phys. Chem..

[20] Sabnis KD (2015). Water-gas shift catalysis over transition metals supported on molybdenum carbide. J. Catal..

[21] Sattler JJ, Ruiz-Martinez J, Santillan-Jimenez E, Weckhuysen BM (2014). Catalytic dehydrogenation of light alkanes on metals and metal oxides. Chem. Rev..

[22] Cui Z (2014). Synthesis of structurally ordered Pt_3_Ti and Pt_3_V nanoparticles as methanol oxidation catalysts. J. Am. Chem. Soc..

[23] Saravanan G (2010). Pt_3_Ti nanoparticles: fine dispersion on SiO_2_ supports, enhanced catalytic CO oxidation, and chemical stability at elevated temperatures. Langmuir.

[24] Naguib M (2013). New two-dimensional niobium and vanadium carbides as promising materials for Li-ion batteries. J. Am. Chem. Soc..

[25] Mashtalir O (2013). Intercalation and delamination of layered carbides and carbonitrides. Nat. Commun..

[26] Lin H, Gao S, Dai C, Chen Y, Shi J (2017). A Two-dimensional biodegradable niobium carbide (MXene) for photothermal tumor eradication in NIR-I and NIR-II biowindows. J. Am. Chem. Soc..

[27] Nawaz Z (2015). Light alkane dehydrogenation to light olefin technologies: a comprehensive review. Rev. Chem. Eng..

[28] Gallagher JR (2015). Structural evolution of an intermetallic Pd-Zn catalyst selective for propane dehydrogenation. Phys. Chem. Chem. Phys..

[29] Wu Z (2016). Pd-In intermetallic alloy nanoparticles: highly selective ethane dehydrogenation catalysts. Catal. Sci. Technol..

[30] Wegener EC (2017). Structure and reactivity of Pt-In intermetallic alloy nanoparticles: Highly selective catalysts for ethane dehydrogenation. Catal. Today.

[31] Hammer B, Morikawa Y, Nørskov JK (1996). CO chemisorption at metal surfaces and overlayers. Phys. Rev. Lett..

[32] Cybulskis VJ (2017). Zinc Promotion of platinum for catalytic light alkane dehydrogenation: insights into geometric and electronic effects. ACS Catal..

[33] Hammer B, Nørskov JK (2000). Theoretical surface science and catalysis-calculations and concepts. Adv. Catal..

[34] Yu W, Porosoff MD, Chen JG (2012). Review of Pt-based bimetallic catalysis: from model surfaces to supported catalysts. Chem. Rev..

[35] Cortright R, Dumesic J (1994). Microcalorimetric, spectroscopic, and kinetic studies of silica supported Pt and Pt/Sn catalysts for isobutane dehydrogenation. J. Catal..

[36] Yang, C. et al. Promotion of Pd nanoparticles by Fe and formation of a Pd_3_Fe intermetallic alloy for propane dehydrogenation. *Catal. Today*, 10.1016/j.cattod.2018.07.043 (2018).

[37] Yang ML, Zhu YA, Zhou XG, Sui ZJ, Chen D (2012). First-principles calculations of propane dehydrogenation over PtSn catalysts. Acs Catal..

[38] Yang ML (2011). DFT study of propane dehydrogenation on Pt catalyst: effects of step sites. Phys. Chem. Chem. Phys..

