# Supplementary material for: Two-dimensional transition metal carbides as supports for tuning the chemistry of catalytic nanoparticles
Source: Nat Commun. 2018 Dec 10;9:5258. doi: 10.1038/s41467-018-07502-5 (PMC6288105; doi:10.1038/s41467-018-07502-5)
Supplement: Supplementary file 1 — Supplementary Information [file 41467_2018_7502_MOESM1_ESM.pdf]

Two-Dimensional Transition Metal Carbides (MXenes) as Supports for Tuning the Chemistry of Catalytic Nanoparticles

Zhe Li et al.

Supplementary Methods

Aberration-corrected scanning transmission electron microscopy

Aberration-corrected imaging and EDS were conducted on a Titan Themis 300 probe corrected TEM equipped with a SuperX EDX detector at Sensitive Instrument Facility (SIF) of Ames Lab. Samples were ground to fine powders and dispersed in isopropyl alcohol. Three drops of the solution or supernatant were added onto an ultrathin carbon lacey film-Au (or Cu) TEM grid (TedPella) and dried naturally. STEM images were taken using the high angle annular dark field (HAADF) detector at 200 kV and particle size was counted using the Gatan Digital Micrograph. A minimum of 100 particles were counted to obtain the size distribution for each catalyst.

X-ray photoelectron spectroscopy (XPS)

XPS data was obtained using a Kratos Axis Ultra DLD spectrometer with monochromic Al Kα radiation (1486.6 eV) at pass energy of 20 and 160 eV for high-resolution and survey spectra, respectively. A commercial Kratos charge neutralizer was used to avoid non-homogeneous electric charging of non-conducting powders and to achieve better resolution. The resolution measured as full width at half maximum of the curve fitted C 1s peak was approximately 1 eV. Binding energy (BE) values refer to the Fermi edge and the energy scale was calibrated using Au $4f_{7/2}$ at 84.0 eV and Cu $2p_{3/2}$ at 932.67 eV. XPS data were analyzed with CasaXPS software version 2313 Dev64. Curve-fitting was performed following a linear or Shirley background subtraction using Gaussian/Lorentzian peak shapes (GL and LF). The atomic concentrations of the elements in the near-surface region were estimated considering the corresponding Scofield atomic sensitivity factors and inelastic mean free path (IMFP) of photoelectrons using standard procedures in the CasaXPS software. For XPS measurements, sample treatments were performed in a reaction cell (≈30 cm^3) connected to the XPS spectrometer and all samples were reduced in 5% H_2 at least for 30 minutes. Then the samples were moved between the reaction cell and the analysis chamber under ultrahigh vacuum (UHV) conditions without contact to air.

In situ X-ray absorption spectroscopy (XAS)

X-ray absorption measurements were acquired at the Ti K edge (4.9664 keV), Nb K edge (18.9856 keV) and Pt L_{III} edge (11.5640 keV) on the insertion device beam line of the Materials Research Collaborative Access Team (MRCAT) at the Sector 10 in the Advanced Photon Source, Argonne National Laboratory. The ionization chambers were optimized for the maximum current with linear response with 10% absorption in the incident ion chamber and 70% absorption in the transmission detector. A third detector in series simultaneously collected a Ti, Nb or Pt metal foil reference spectrum with each measurement for energy calibration. Solid samples were pressed into a cylindrical sample holder consisting of six wells, forming a self-supporting wafer. The sample holder was placed in a quartz reactor tube sealed with Kapton windows by two Ultra-Torr fittings through which gas could be flowed.

$Ti_3C_2T_x$ and reference compounds TiO_2, Ti foil and TiC (Sigma-Aldrich) were scanned in air. Fresh Pt on $Ti_3C_2T_x$ and Nb_2CT_x catalysts were reduced in 3 % H_2/He with a flow rate of 50 cm^3/min at 550 °C for at least 30 min, then cooled to room temperature and flushed with He or air before they were scanned. The fits of the Extended X-ray Absorption Fine Structure (EXAFS) were evaluated using Artemis software (Demeter 0.9.26). The EXAFS coordination parameters were obtained by a least-squares fit in R-space of the k^2-weighted Fourier transform data from 3.0 to 11.0 $Å^{-1}$ and the first shell fits of the magnitude and imaginary parts were performed between 1.0 and 2.2 Å.

Intercalation and delaminated of $Ti_3C_2T_x$ and Nb_2CT_x

Around 0.1 g of the dried $Ti_3C_2T_x$ or Nb_2CT_x MXene powders was dispersed into ~5ml dimethyl sulfoxide and tetrapropylammonium hydroxide. The solutions were then stirred at ambient temperature for 24 hours. The resulting solutions were centrifuged @ 8900 rpm to separate the precipitates from the solvents and deionized water was added to the powders in a weight ratio of MXene to water of 1:200. Then, the suspensions were sonicated in a bath for 0.5 hour and the supernatant was collected for further TEM characterizations. Finally, the MXene powders were washed twice using ethanol to completely remove the organic intercalants and collected by centrifugation and dried in vacuum at room temperature overnight.

Determination of the platinum loading of Pt/MXene catalysts

Pt loadings of the Pt/$Ti_3C_2T_x$ and Pt/Nb_2CT_x catalysts were determined by atomic absorption spectroscopy (AAS). Specifically, the catalyst was digested by aqua regia in a Nalgene® amber polyethylene bottle for 3 days and the solution was then diluted to desired concentration for the AAS measurement.

Computational methods

Density functional theory (DFT) calculations were performed using the plane-wave based PWSCF (Quantum-ESPRESSO) program and the Atomic Simulation Environment (ASE)[1]. The ultrasoft Vanderbilt pseudopotential method with Perdew-Burke-Ernzerhof (PBE) exchange-correlation functional was adopted[2-4]. A plane-wave cutoff energy of 500 eV was used. The (111) slabs were built with 4 atomic layers in hexagonal 4×4 supercells with the bottom two layers fixed during structural relaxation. The Monkhorst-Pack scheme was used for sampling the Brillouin zone and k-point grids of 2×2×1 were selected[5]. The vacuum thickness was set to 15 Å in all slab calculations and the dipole correction was applied to decouple the interaction between slabs. During structural optimizations, the residual force between atoms was converged to a value below 0.02 eV/Å. The free energies of reaction intermediates and transition states on the surface were calculated as E_{total} + ZPE + H_{vib} − TS_{vib}, where E_{total} is DFT calculated total energy, ZPE is the zero point energy, T is temperature, H_{vib} and S_{vib} are the enthalpy and entropy parts from non-imaginary vibrations derived in a harmonic approximation to the potential. The free energies of gas phase molecules were calculated as E_{total} + ZPE + H − TS, where H is the enthalpy and S is the entropy. Transition states were calculated using the Fixed Bond Length method as implemented in the ASE.

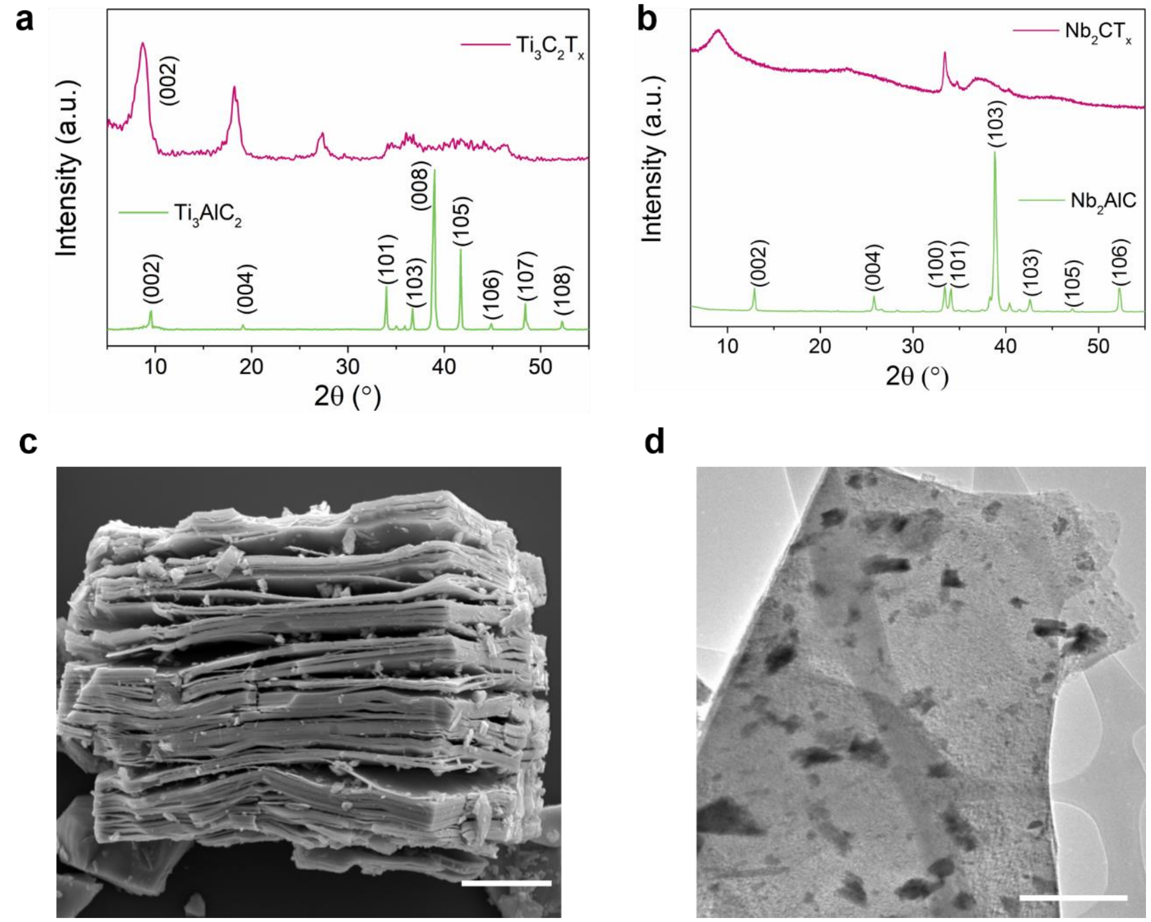

Supplementary Figure 1

(a) and **(b)** XRD patterns of Ti_3AlC_2, $Ti_3C_2T_x$ MXene, Nb_2AlC and Nb_2CT_x MXene. The downshifts of (002) peaks to lower angles indicate that the MAX phases have converted to the corresponding MXenes. **(c)** SEM image of Nb_2CT_x MXenes. The MXene show the typical ‘‘accordion’’ morphology, confirming the exfoliation of individual grain along the basal planes. **(d)** Bright field TEM image of Nb_2CT_x MXene. Scale bars are 3μm.

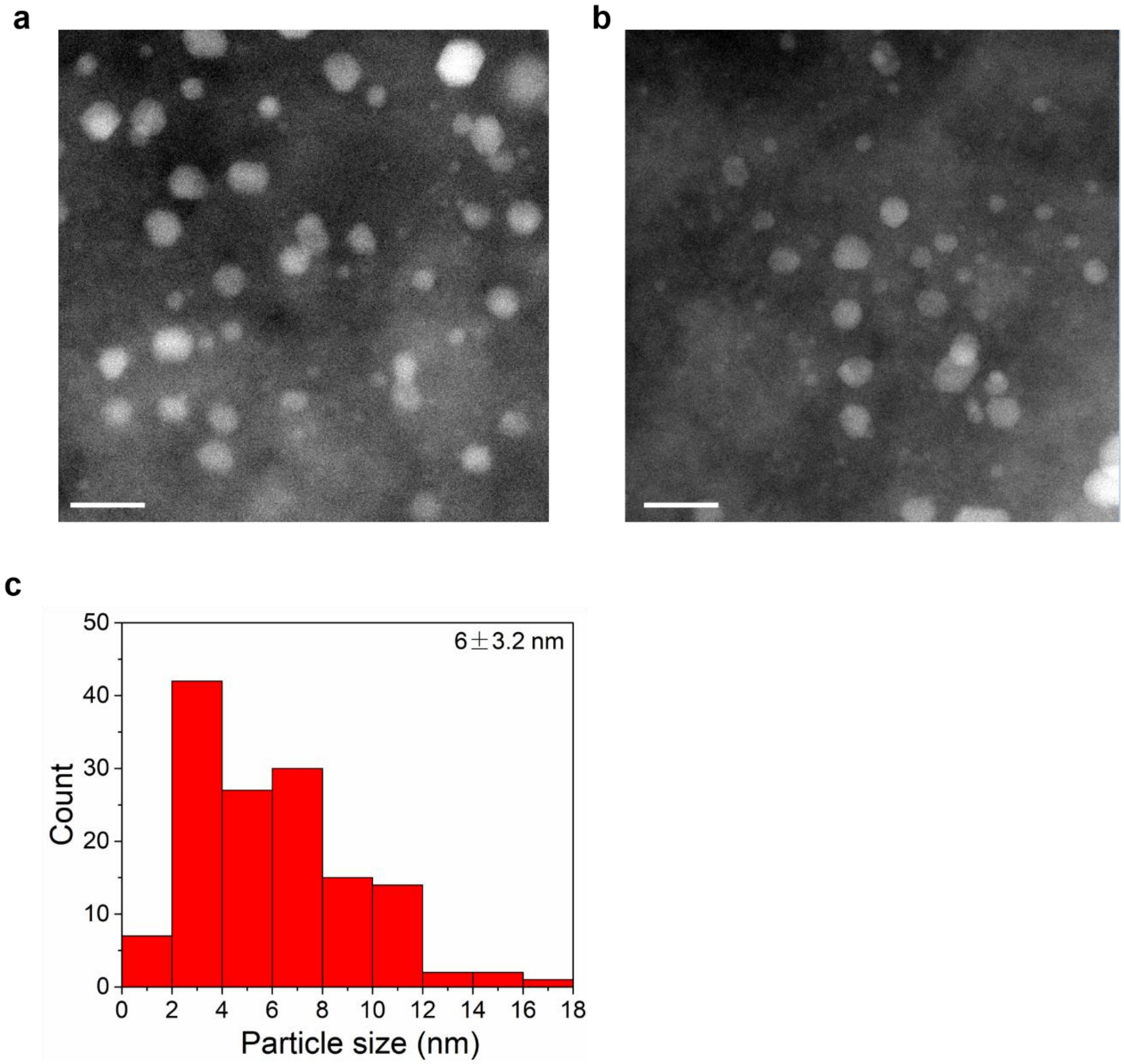

Supplementary Figure 2
(a) and **(b)** Annular dark-field STEM overview image of Pt/$Ti_3C_2T_x$ reduced by 5% H_2/N_2 at 550 °C. Scale bars correspond to 20 nm. **(c)** Particle size distribution of more than 100 particles. The average particle size is 6 nm.

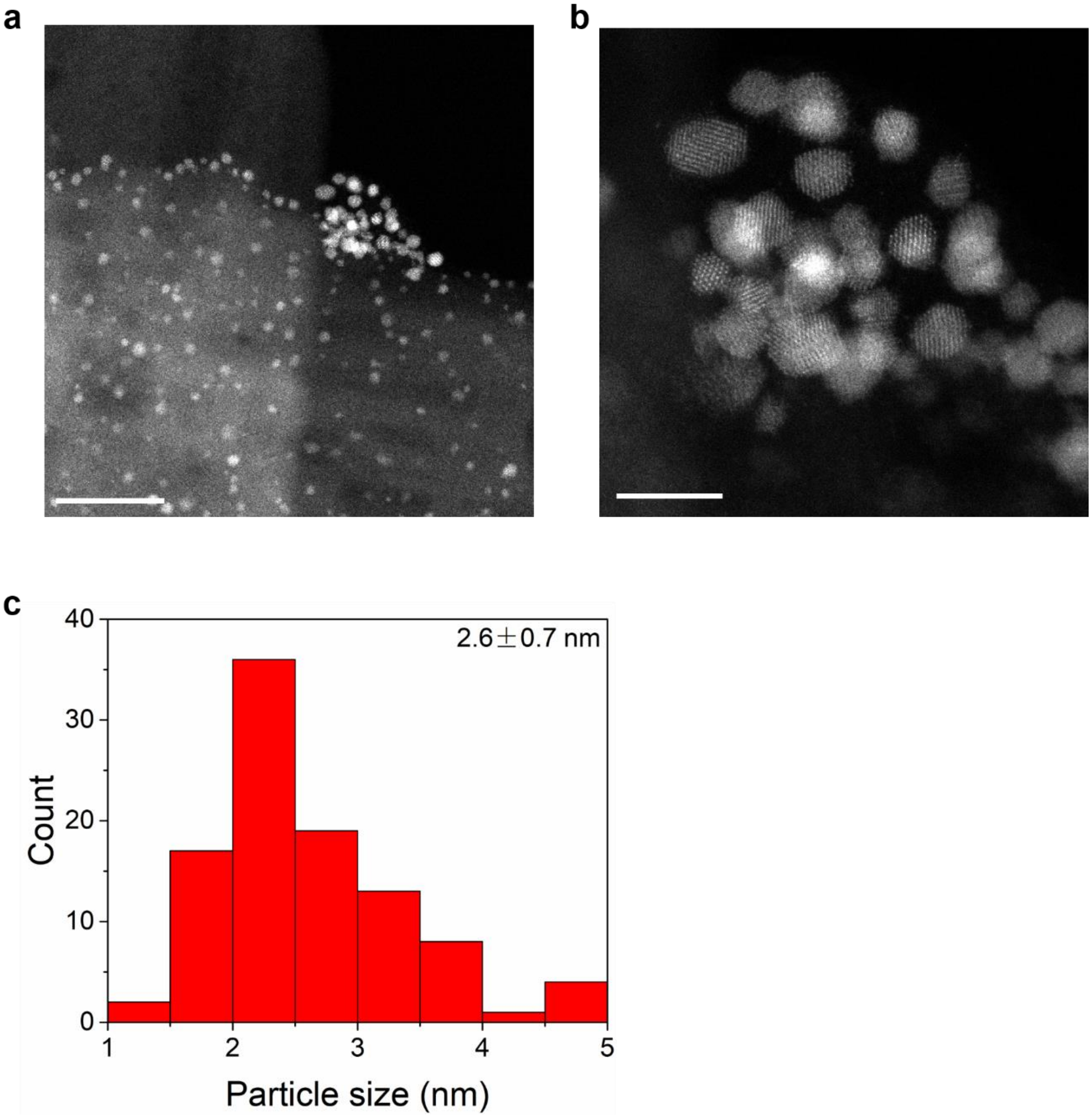

Supplementary Figure 3

(a) and **(b)** Annular dark-field STEM overview image of Pt/Nb_2CT_x reduced by 5% H_2/N_2 at 550 °C. Scale bars correspond to 20 nm and 5 nm, respectively. **(c)** Particle size distribution of more than 100 particles. The average particle size is 2.6 nm.

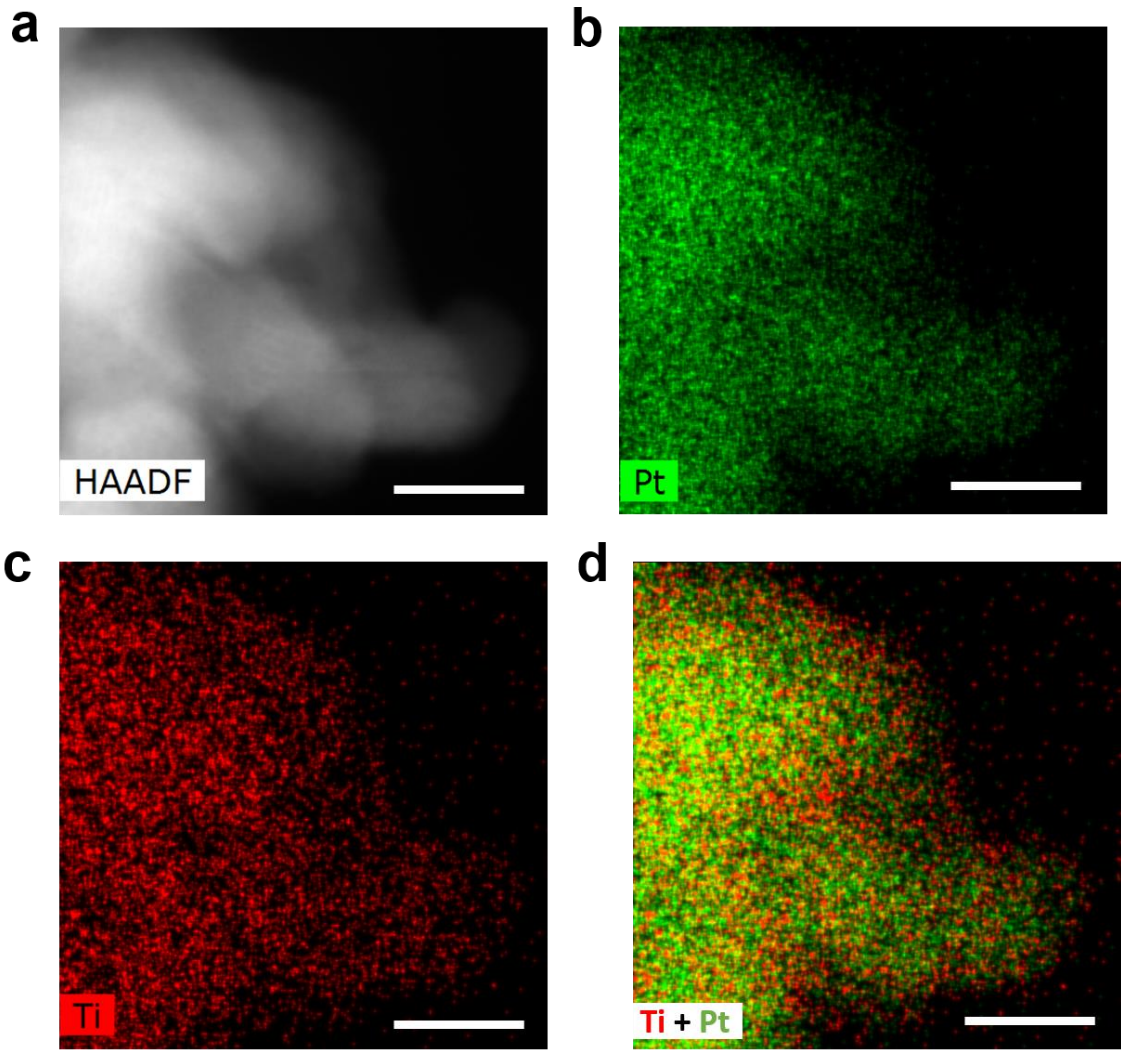

Supplementary Figure 4
(a) HAADF-STEM image of Pt/$Ti_3C_2T_x$. **(b-d)** Elemental mappings of Pt **(b)**, Ti **(c)**, and Ti versus Pt **(d)**. Scale bars are 4 nm.

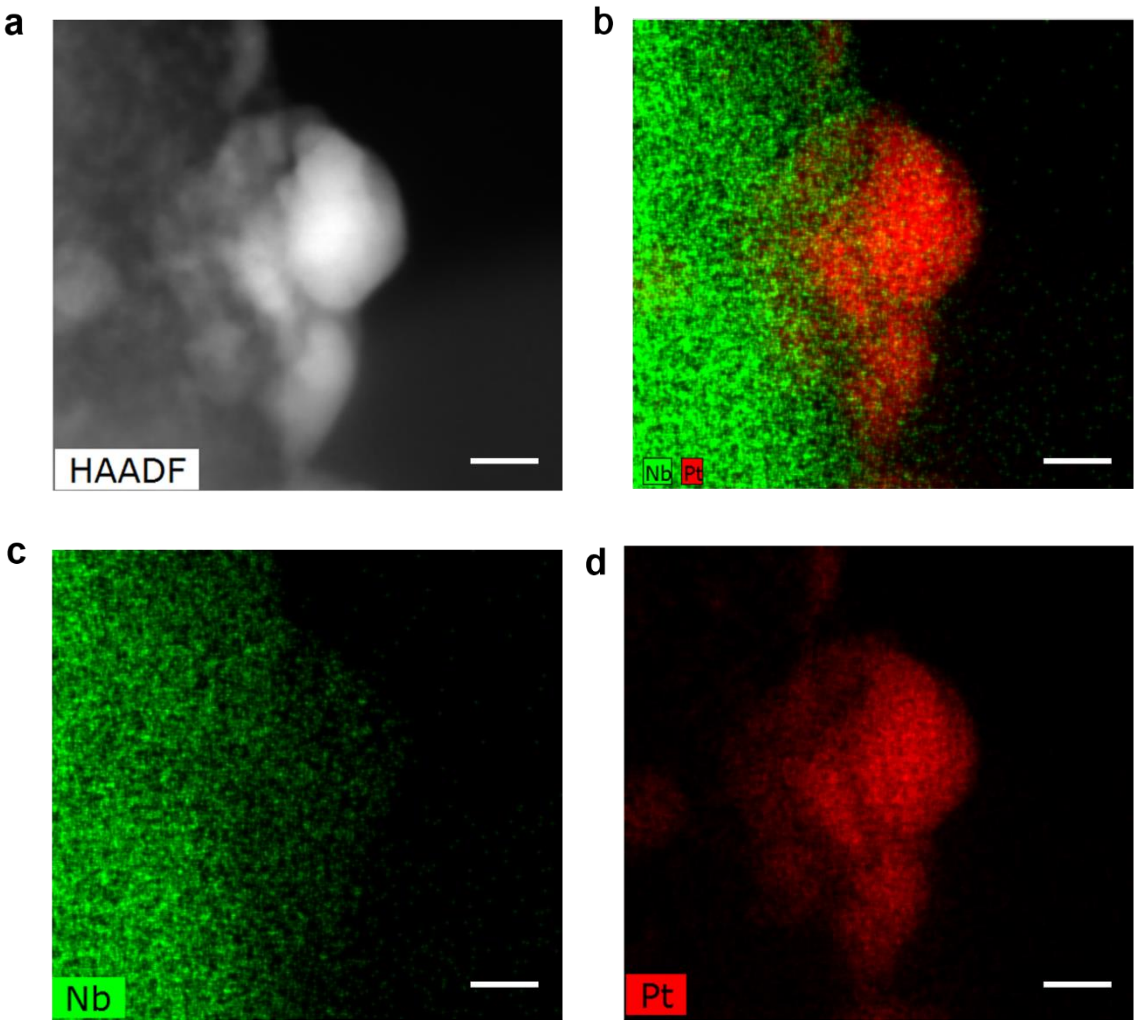

Supplementary Figure 5

(a) HAADF-STEM image of Pt/Nb_2CT_x. **(b-d)** Elemental mappings of Nb versus Pt **(b)**, Nb **(c)**, and Pt **(d)**. Scale bar, 5 nm.

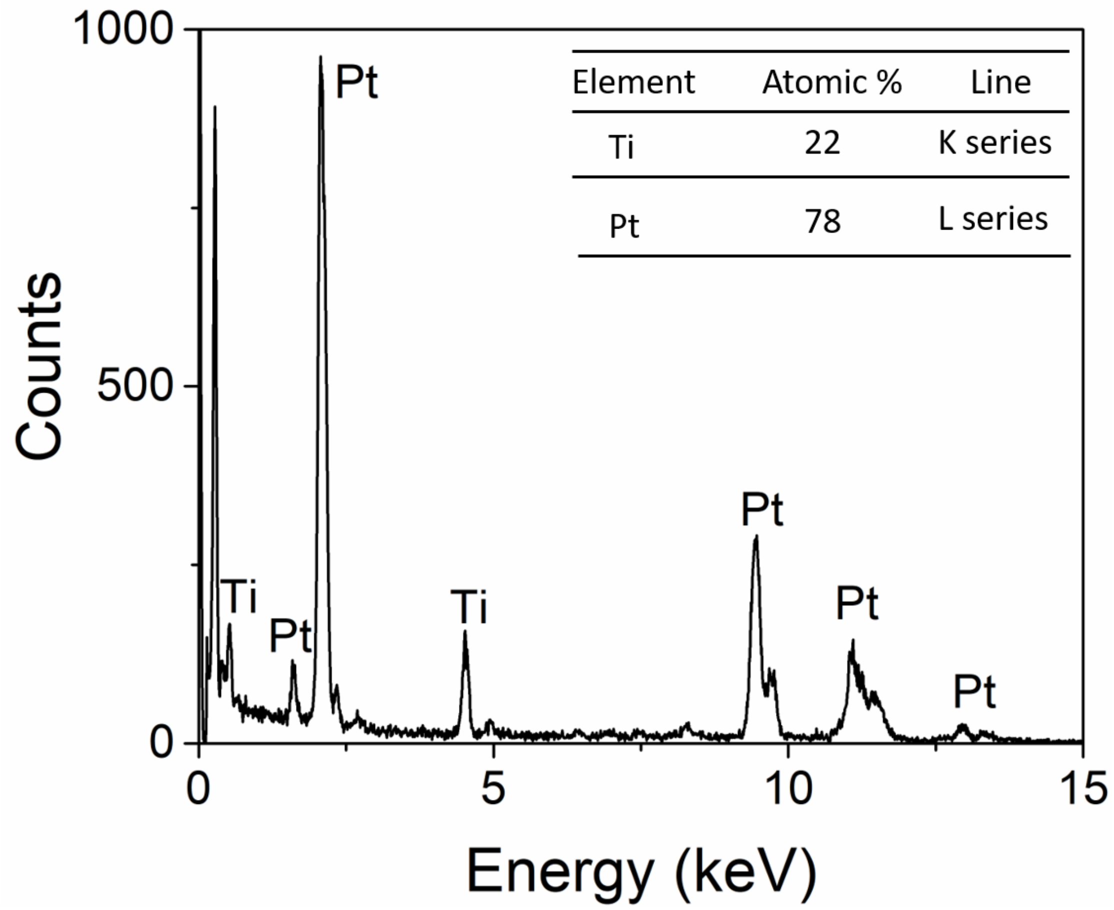

Supplementary Figure 6
Energy dispersive X-ray spectroscopy (EDS) signal counts of Pt-Ti alloy shown in supplementary Fig. 4. The inset shows the atomic percentage of Ti and Pt. The Pt:Ti molar ratio is 3.55.

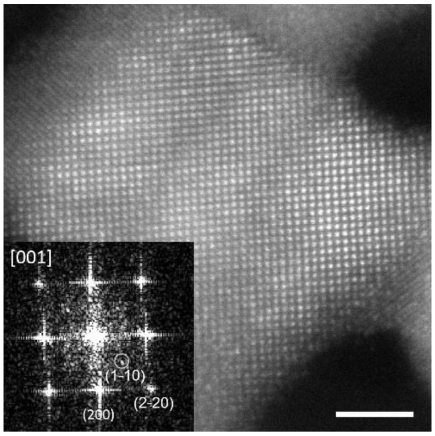

Supplementary Figure 7

A Pt-Nb NP viewed along [001]. Inset is the FFT pattern of the NP, which shows the signals of the (1-10) superlattices. The scale bar corresponds to 2 nm

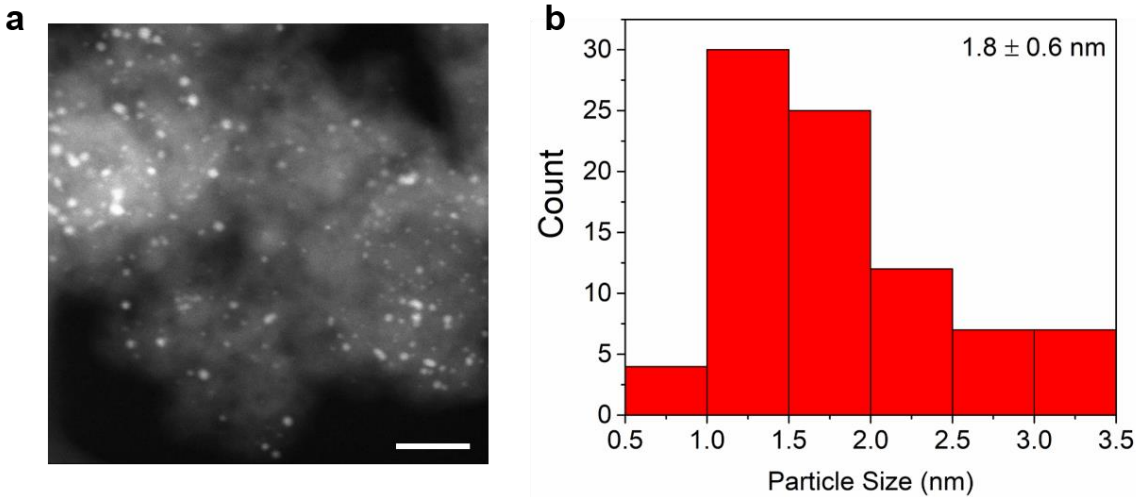

Supplementary Figure 8

(a) Annular dark-field STEM overview image of Pt/SiO_2 reduced by 5% H_2/N_2 at 550 °C. The scale bar corresponds to 20 nm. **(b)** Particle size distribution of Pt/SiO_2. The average particle size is 1.8 nm.

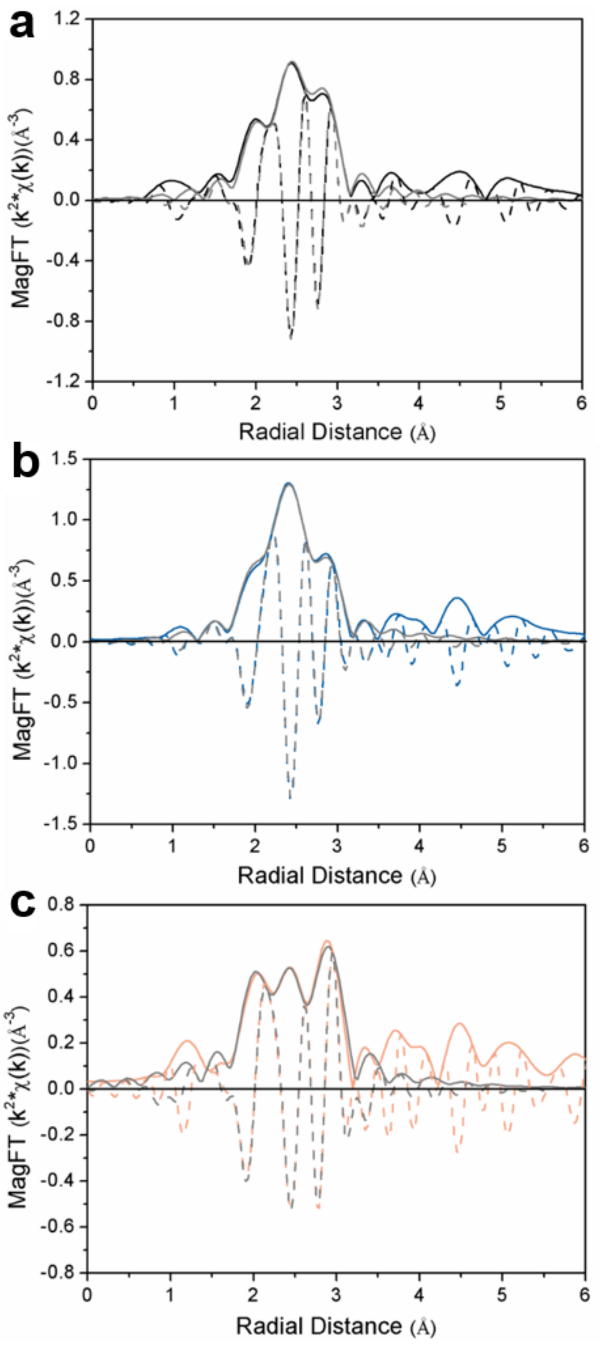

Supplementary Figure 9

The magnitude (solid) and imaginary (dash) part of the Fourier transform of the k^2 weighted EXAFS and corresponding first shell fit (grey) for **(a)** Pt/SiO_2 (black), **(b)** Pt/$Ti_3C_2T_x$ (blue) and **(c)** Pt/Nb_2CT_x (orange). The fitting ranges are $\Delta k = 3.0\text{-}12.2$ Å^{-1} and $\Delta R = 1.6\text{-}3.2$ Å. Corresponding fitting results are listed in Supplementary Table 1.

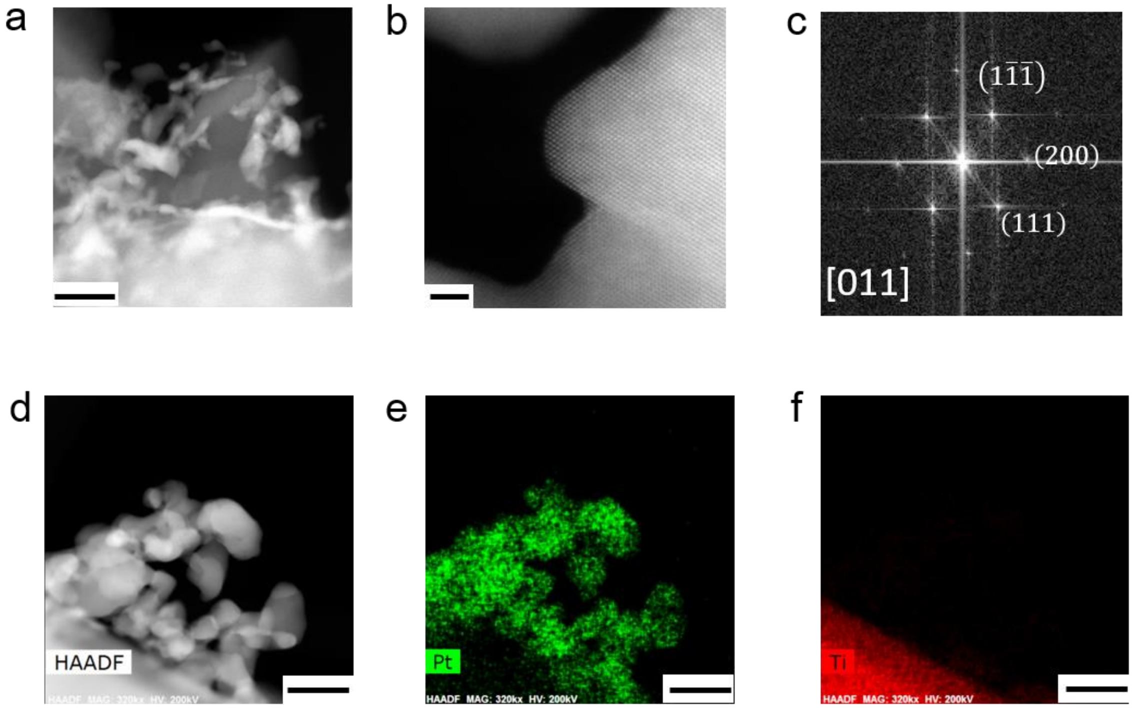

Supplementary Figure 10

Pt/TiC (bulk carbide) sample reduced at 550°C by 4% H_2. (a) HAADF-STEM image of Pt/TiC. (b-c) atomic resolution HAADF-STEM image and corresponding FFT patterns of Pt/TiC. (d-f) EDS analysis of Pt/TiC. Sale bars: (a), (d), (e), (f) 50 nm, and (b) 2 nm.

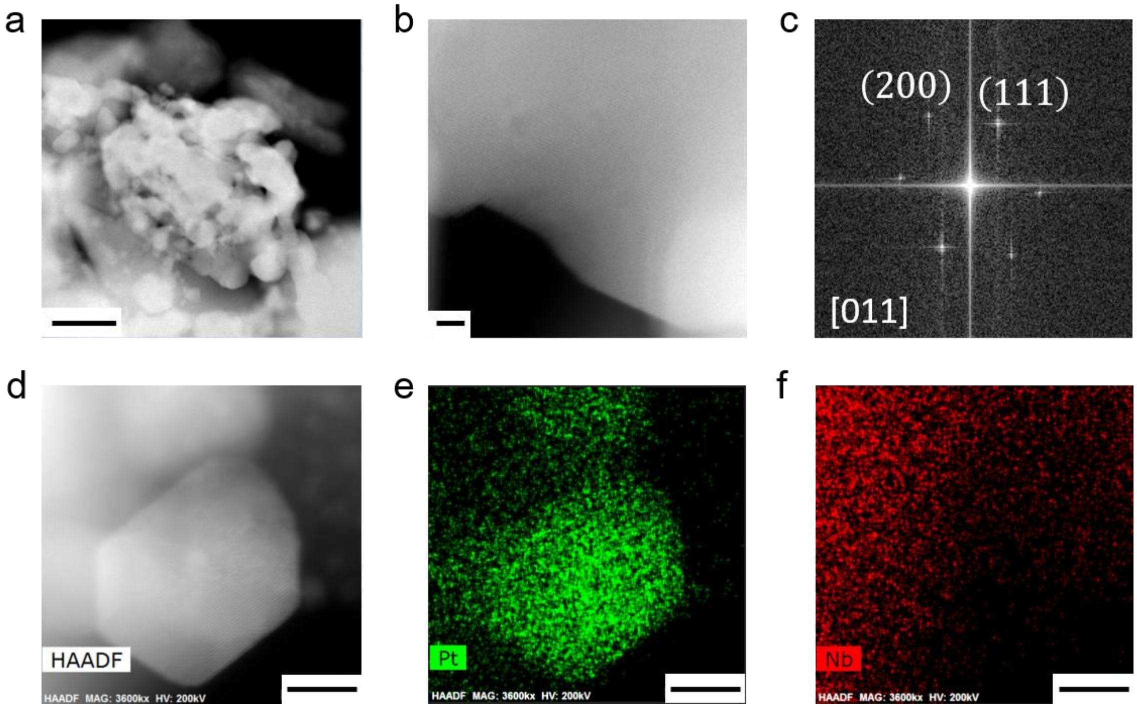

Supplementary Figure 11

Pt/NbC (bulk carbide) sample reduced at 550℃ by 4% H_2. (a) HAADF-STEM image of Pt/NbC, the scale bar corresponds to 100 nm. (b-c) atomic resolution HAADF-STEM image and corresponding FFT patterns of Pt/NbC, the scale bar in (b) is 2 nm. (d-f) EDS analysis of Pt/NbC, scale bars 5 nm.

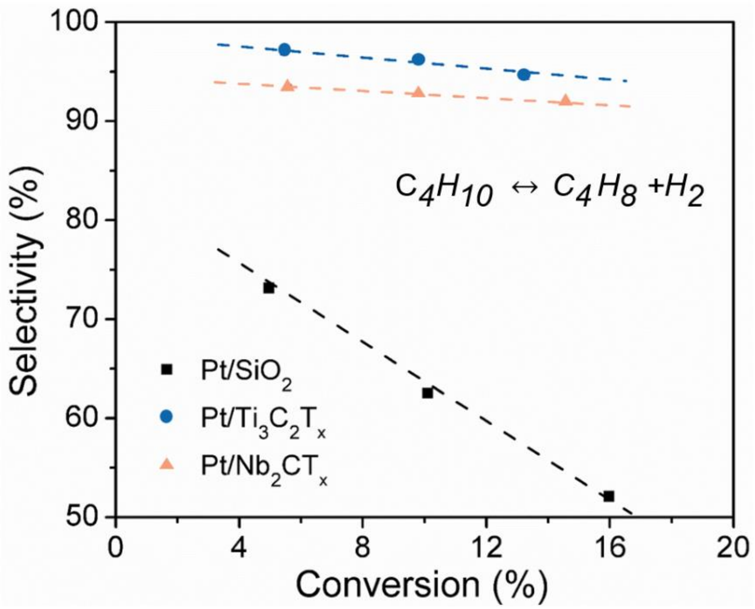

Supplementary Figure 12

Plots of conversion *vs.* selectivity of iso-butane dehydrogenation measured in 100 cm^3/min of 2.5 % C_4H_{10}, 2.5 % H_2 balanced in N_2 at 1.5 atm and 450 °C for Pt/ $Ti_3C_2T_x$, Pt/Nb_2CT_x and Pt/SiO_2 catalysts.

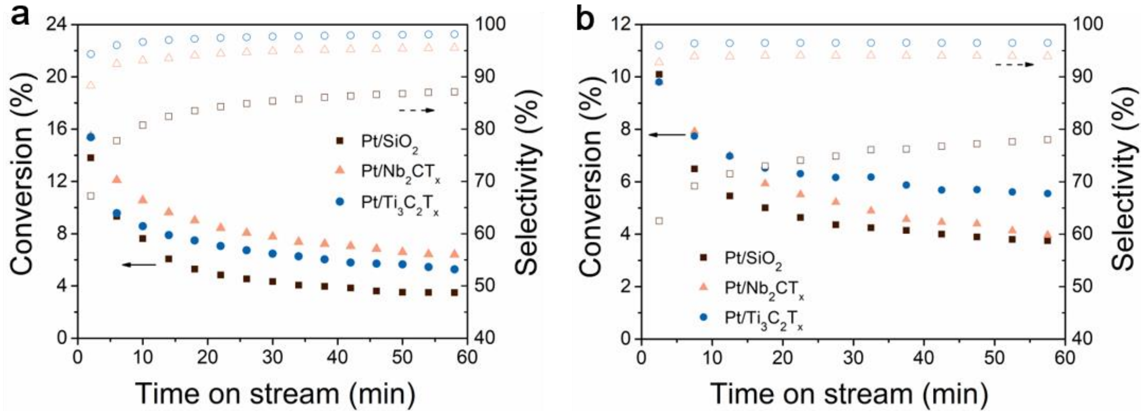

Supplementary Figure 13

(a) Time on stream of propane dehydrogenation reaction over Pt/MXenes and Pt/SiO_2 catalysts. (b) Time on stream of isobutane dehydrogenation reaction over Pt/MXenes and Pt/SiO_2 catalysts.

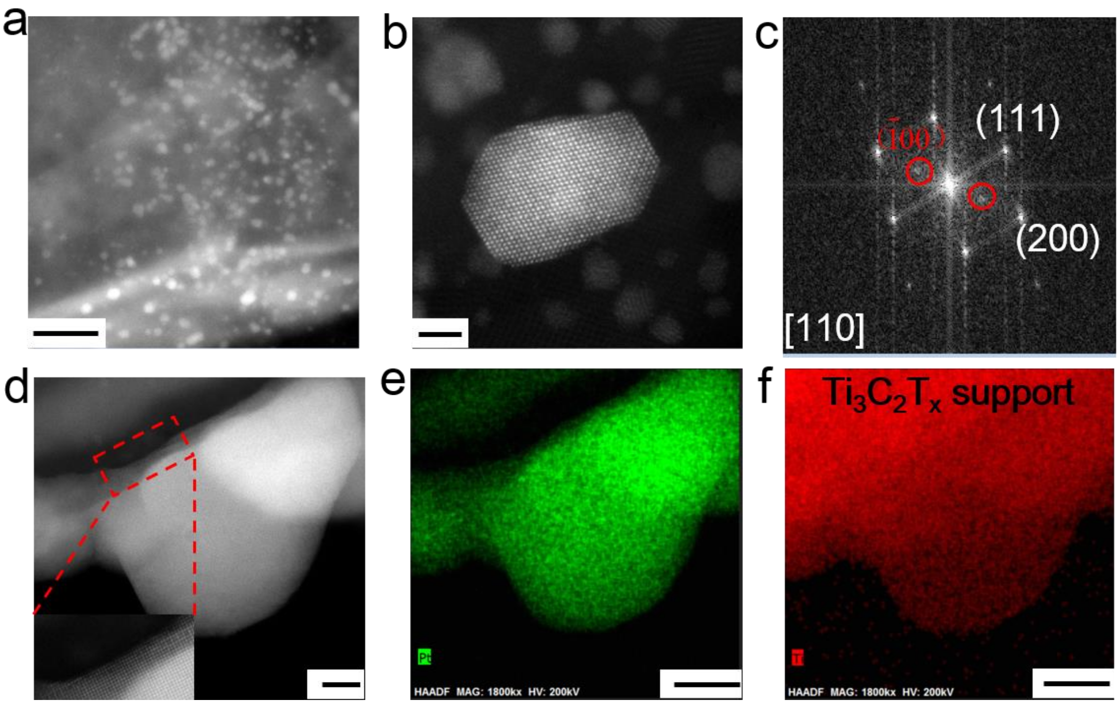

Supplementary Figure 14

(a) HAADF-STEM image on post PDH Pt/$Ti_3C_2T_x$ catalyst, the scale bar corresponds to 50 nm. **(b-c)** Atomic resolution HAADF-STEM image and FFT pattern of Pt_3Ti NP, the scale bar in (b) is 2 nm. (d-f) EDS analysis of used Pt/$Ti_3C_2T_x$ catalyst, scale bars: (d) 5nm, (e) and (f) 9 nm.

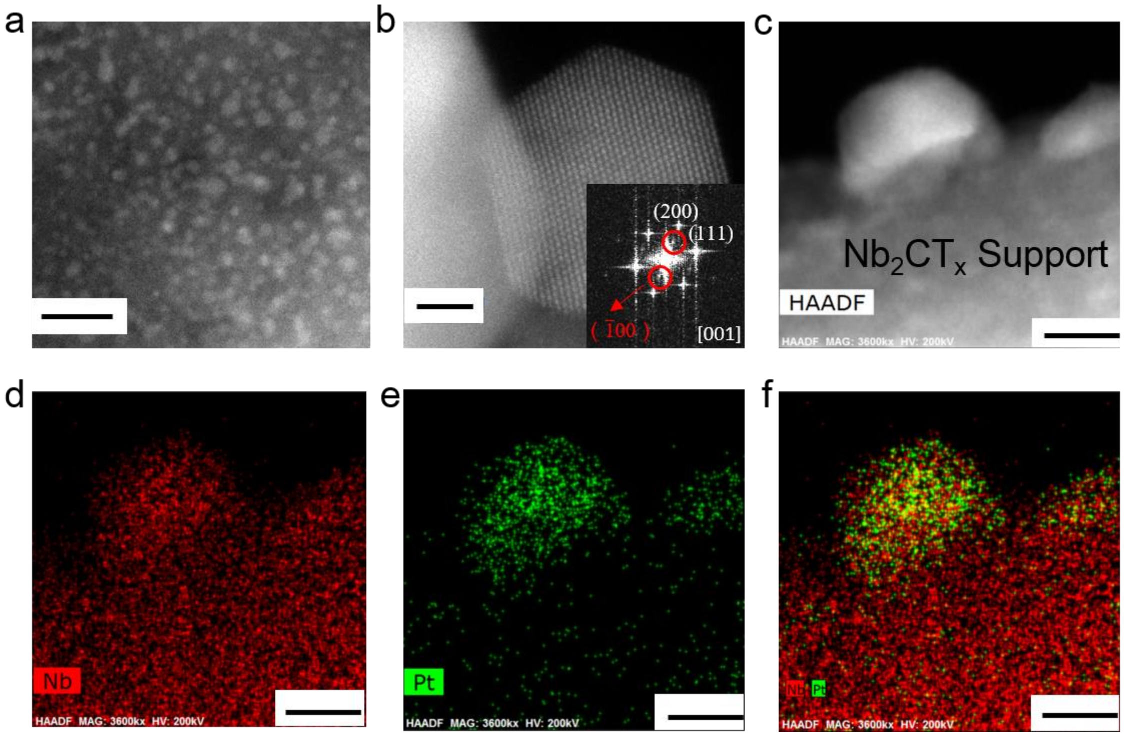

Supplementary Figure 15

(a) HAADF-STEM image on post PDH Pt/Nb_2CT_x catalyst, scale bar 10 nm. **(b)** Atomic resolution HAADF-STEM image and FFT pattern of Pt_3Nb NP, scale bar 2 nm. (c-f) EDS analysis of used Pt/Nb_2CT_x catalyst, scale bars 5 nm.

Supplementary Table 1.

Quantitative information of the EXAFS fits (Figure S9)

Sample	Scattering Pair	S_0^2	CN #	r (Å) #	ΔE_0 (eV) #	σ^2 (Å ^{2}) #	R factor
Pt/SiO_2	Pt-Pt	0.77 *	8.6	2.74	7.1	0.006	0.004
Pt/$Ti_3C_2T_x$	Pt-Pt	0.77 *	6.6	2.75	6.2	0.006	0.003
	Pt-Ti		3.4	2.75		0.009	
Pt/Nb_2CT_x	Pt-Pt	0.77 *	6.7	2.77	5.5	0.006	0.011
	Pt-Nb		1.8	2.75		0.008	

* The S_0^2 is fixed at the value obtained by fitting a Pt foil reference.

# The error of all the fitted parameters are close. The average error in CN (coordination number) is 1, in r (bond length) is 0.01 Å, in ΔE_0 (energy shift) is 1.0 eV and in σ^2 (Debye-Waller factor) is 0.001 Å^2.

Supplementary References

1 Paolo, G. *et al.* QUANTUM ESPRESSO: a modular and open-source software project for quantum simulations of materials. *J. Phys.: Condens. Matter* **21**, 395502 (2009).

2 Laasonen, K., Car, R., Lee, C. & Vanderbilt, D. Implementation of ultrasoft pseudopotentials in ab initio molecular dynamics. *Phys. Rev. B* **43**, 6796-6799 (1991).

3 Laasonen, K., Pasquarello, A., Car, R., Lee, C. & Vanderbilt, D. Car-Parrinello molecular dynamics with Vanderbilt ultrasoft pseudopotentials. *Phys. Rev. B* **47**, 10142-10153 (1993).

4 Perdew, J. P., Burke, K. & Ernzerhof, M. Generalized gradient approximation made simple. *Phys. Rev. Lett.* **77**, 3865-3868 (1996).

5 Monkhorst, H. J. & Pack, J. D. Special points for Brillouin-zone integrations. *Phys. Rev. B* **13**, 5188-5192 (1976).
